# Reward and empathy in the treating clinician: the neural correlates of successful doctor–patient interactions

**DOI:** 10.1038/s41398-020-0712-2

**Published:** 2020-01-21

**Authors:** Karin Jensen, Randy L. Gollub, Jian Kong, Claus Lamm, Ted J. Kaptchuk, Predrag Petrovic

**Affiliations:** 1 Department of Clinical Neuroscience, Karolinska Institutet, Stockholm, Sweden; 2 Department of Psychiatry and Martinos Center for Biomedical Imaging, Massachusetts General Hospital, Harvard Medical School, Boston, MA USA; 3 Social, Cognitive and Affective Neuroscience Unit, Department of Basic Psychological Research and Research Methods, Faculty of Psychology, University of Vienna, Vienna, Austria; 4 Program in Placebo Studies, Beth Israel Deaconess Medical Center, Harvard Medical School, Boston, MA USA

**Keywords:** Human behaviour, Learning and memory

## Abstract

The goal of this study was to determine the neural correlates of successful doctor–patient interactions. We performed an experimental neuroimaging study where medical doctors (MDs) performed a treatment task while their brain activation pattern was measured, using functional magnetic resonance imaging (fMRI). MDs (25–37 years old) first performed a standardized clinical exam of a “professional patient”. Unbeknownst to the doctors, the professional patient was a confederate that rated the doctors’ clinical examination using the Consultation And Relational Empathy (CARE) questionnaire, a standardized protocol assessing a clinician’s social interaction during a consultation. After the clinical exam, MDs were placed inside a brain scanner and the patient was placed on a chair next to the MD. MDs performed a treatment task where an analgesic device was used to alleviate the patient’s pain (experimentally induced), while the MD’s brain activity was measured with fMRI. MDs rated their own empathic concern (equivalent of compassion) and personal distress using the Interpersonal Reactivity Index questionnaire. The patient’s rating of CARE was robustly related to the MD’s own ratings of trait empathic concern and to compassion-related and reward-related activation of medial frontal brain regions during treatment. In contrast, there was no relation with MD’s personal distress, nor with activation in regions associated with the aversive component of experiencing empathy. We conclude that a patient’s positive experience of a medical examination is reflected in doctors’ empathic concern and reward-related brain activations during treatment, suggesting that compassion and pleasure are key factors for successful doctor–patient interactions.

## Introduction

Physicians treating patients are potentially exposed to two opposite psychological processes: one positive feeling related to the experience of helping someone in need, and, on the other hand, the aversive experience related to witnessing someone’s suffering or frustration at the inability to help. The ability to share the feelings of others is often referred to as empathy, and the ability to care for and show concern for others is the core aspect of compassion^1–3^.

The affective state of the professional health worker is of importance for improvement of medical treatments since the clinical dyad between the patient and the clinician has direct consequences for treatment outcomes^4,5^. While clinicians’ ability to experience empathy in general has been suggested to be an important factor for successful treatment^6^, it has also been suggested that some aspects of empathy may be maladaptive since it might result in aversive responses, including personal distress in the clinician^1,6^. In fact, affective burnout is a major cause for sick-leave in doctors^7^. Researchers have suggested that compassion processes, involving the brain’s reward circuitry, may be a strategy for interacting with patients^1^. While the distressful component of empathy (often denoted as personal distress^8^) and compassion may reflect different affective states of the clinician (and thereby shape the interaction with the patient), there is little empirical evidence as to which strategy is more critical and leads to better outcomes for the patient. Empathic concern^8^ is another term used interchangeably for compassion or sympathy, but it should not be confounded with empathy in the sense of negative affect sharing^2,3^. While empathic concern, sympathy and compassion represent “other-oriented” responses to someone suffering, the personal distress component of empathy is a “self-oriented” response^9^.

Distinct neural correlates associated with personal distress and compassion have been demonstrated in various paradigms when viewing individuals in pain^1,10–14^, including physicians while they treat^15^. Thus, it is possible to explore if and how these two separate processes are active during patient–physician interactions. In an experimental setting, we studied whether a person’s rating of a medical exam was related to personal distress or compassion (empathic concern) in the treating physician. This novel analysis was based on our previous study where medical doctors underwent brain scanning during a treatment task^15^. The results presented here are exploratory analyses in addition to the primary results in our previous paper.

## Materials and methods

In the present study 18 physicians (aged 25–37 years; ten female) participated after written consent was given. The physicians performed (what they believed to be real transcutaneous electrical nerve stimulation [TENS]) treatment of pain to the arm in one of two professional patients—i.e., confederates getting sham TENS treatment (25-year-old female research assistants, here referred to as “patients”)—while undergoing functional magnetic resonance imaging (fMRI). From the same experiment, we have previously reported activations related to expectations, empathy-processing and reward-processing during treatment^15^. Here, we focused on physician brain activations during treatment in regions observed in “empathy for pain” and compassion studies^1,10–14^, and, most importantly, related this to the patient’s experience of the physicians’ medical exam (as an indicator of successful treatment).

**Fig. 1:**
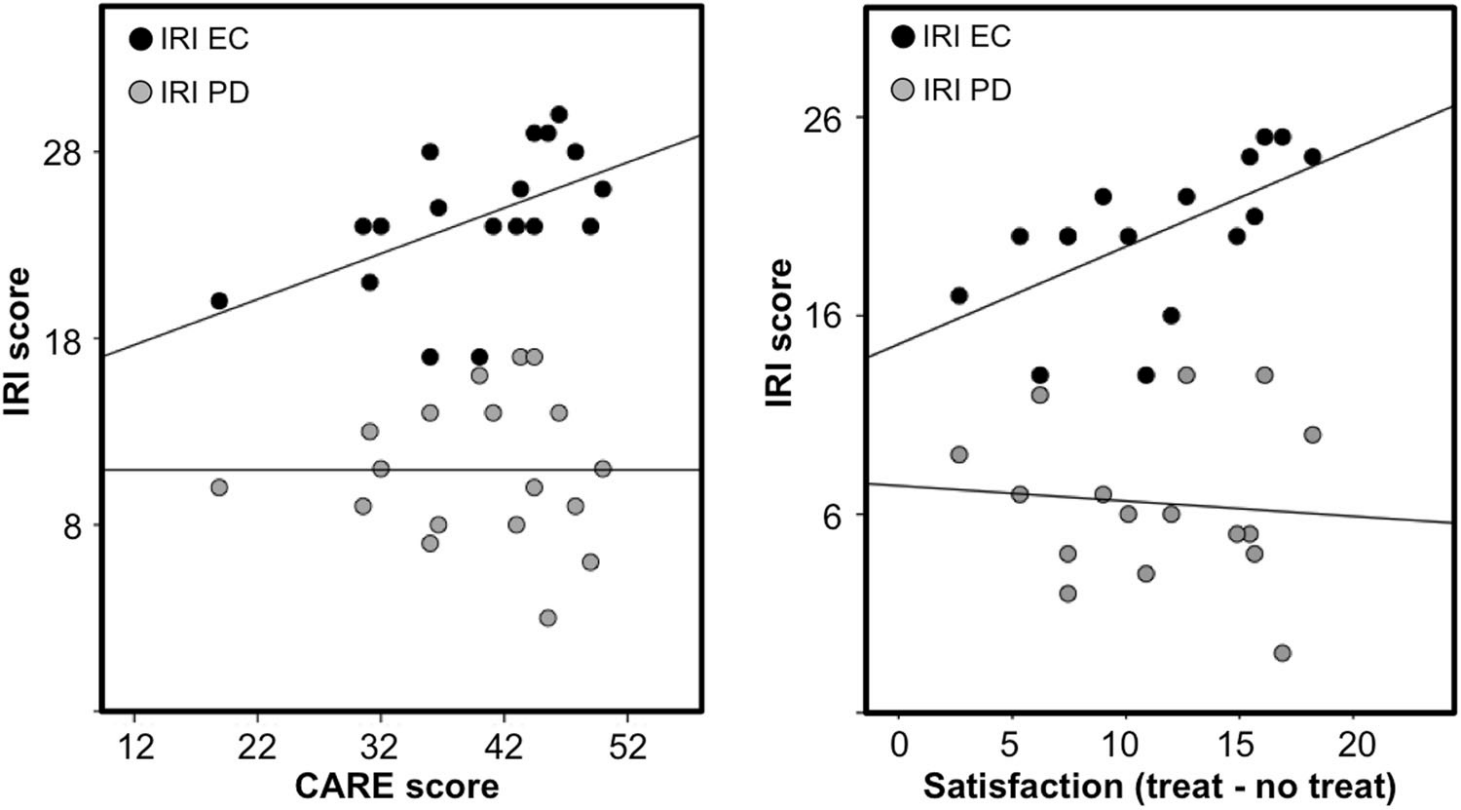
Physician IRI correlations. Left: CARE scores correlated significantly with physicians’ empathic concern (EC) (*r*_s_ = 0.595, *p* = 0.009) but not with personal distress (PD) (*r*_s_ = -0.058, *p* = 0.819). This result survived when outliers were removed. Right: Physician satisfaction scores (rated on a numeric scale from −10 “completely dissatisfied” to +10 “completely satisfied”) during the fMRI pain relief experiment (treatment condition vs. no-treatment condition) correlated significantly with empathic concern (EC) (*r*_s_ = 0.707, *p* = 0.002) but not with personal distress (PD) (*r*_s_ = −0.080, *p* = 0.769). Spearmans rho was used for correlational analyses.

The physicians performed an initial clinical examination of the patients (~20 min) before the experiment. This allowed us to measure how the patients experienced the patient–physician interaction using the validated consultation and relational empathy (CARE) questionnaire after the clinical exam^16^. CARE is a 10-item scale developed to measure the patient’s perception of a clinician’s relational empathy and communication skills during a consultation. The questions assess “other-oriented” behavior of the clinician and reflect empathic concern. We also measured physicians’ empathic concern (EC) and personal distress (PD) of the interpersonal reactivity index (IRI)^8^. While EC assesses other-oriented feelings of sympathy and concern for unfortunate others (closely related to compassion), PD measures self-oriented feelings of personal anxiety and unease in tense personal settings (related to the distressful aspect of empathy)^2,3,8^. Finally, we used physicians’ satisfaction scores to measure state satisfaction during the treatment task (Fig. 1). CARE was not reported in the original article^15^ since it was not related to the main hypothesis. For completeness we also describe which other scales were administered in the original study but not reported: The general scale of self-efficacy (GSSE)^17^, Spielberger state-trait anxiety inventory^18^, as well as a question regarding “satisfaction being an MD” (0–100%), and a question asking for “confidence in ability to build a relationship with patients” (0–100%).

Correlations were performed using Spearman’s Rho (two-sided), as our behavioral data was not normally distributed. Further methodological details are available elsewhere^15^. The fMRI-data was analyzed using Statistical Parametric Modeling 12 (SPM12; Wellcome Trust Center for Neuroimaging, London, UK http://www.fil.ion.ucl.ac.uk/spm). We were specifically interested in regions either belonging to a network processing empathy for pain^12^ or regions associated with compassion training^1^. The empathy for pain related regions included the right anterior insula [rAI] and caudal anterior cingulate cortex [cACC]), whereas the compassion related regions included (1) a region encompassing medial Orbitofrontal cortex [mOfc] and subgenual ACC [sgACC] and (2) a region encompassing rostral ACC [rACC] and ventromedial prefrontal cortex [vmPFC] as well as (3) ventral striatum [VS]. These a priori ROIs were derived from coordinates in Klimecki and Singer^1^ (for compassion training related regions) and Lamm et al.^12^ (for empathy for pain related regions) using 5 mm sphere radius around peak voxel associated regions. The initial statistical threshold for the ROI-analysis was set to *p* < 0.01 (uncorrected). A family-wise error (FWE) correction for multiple comparison (*p* < 0.05) was then performed on voxel and cluster level within the ROIs. The age of the physicians was included as a covariate of no interest in the fMRI analysis.

Ethical approval was received from the Institutional Review Board of the Massachusetts General Hospital and MDs signed an informed consent. After completing the study, MDs were fully debriefed about the confederate “patient”, and the sham device, and offered to withdraw their data. None withdrew their data.

## Results

### Behavioral results

CARE scores showed a moderate and positive correlation with physicians’ self-reported EC (*r*_s_ = 0.595, *p* = 0.009; Fig. 1). While CARE did not significantly correlate with the degree of treatment satisfaction expressed by the physicians (*r*_s_ = 0.141, *p* = 0.602), EC was significantly correlated with treatment satisfaction (change in physician satisfaction after treatment versus no-treatment; *r*_s_ = 0.707, *p* = 0.002). In contrast, CARE showed no correlation with physician’s PD ratings (*r*_s_ = −0.058, *p* = 0.819), and PD did not correlate with treatment satisfaction (*r*_s_ = −0.080, *p* = 0.769).

### Neuroimaging results

**Fig. 2:**
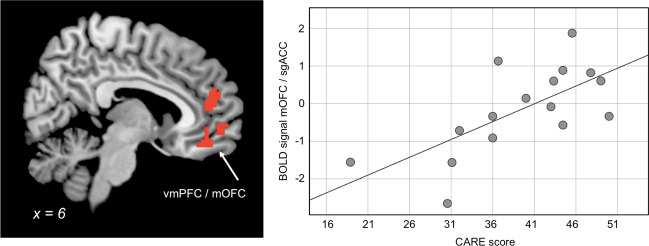
Physician brain activity relating to patient CARE ratings. Left: Brain regions where physicians displayed increased treatment-related activity when patients’ CARE ratings were high included medial orbitofrontal cortex (mOFC)/subgenual anterior cingulate cortex (sgACC) as well as the rostral anterior cingulate cortex (rACC)/ventromedial prefrontal cortex (vmPFC). The initial statistical threshold was *p* < 0.01, uncorrected. A family-wise error (FWE) correction for multiple comparison (*p* < 0.05) was then performed on voxel and cluster level within the a priori ROIs. Right: Scatterplot of CARE scores (*x*-axis) and physicians’ BOLD signal from the mOFC/sgACC (*y*-axis), where activations were high when CARE was high. BOLD signal was extracted from the entire cluster with peak voxel in MNI coordinates *x* = 9, *y* = 41, *z* = −17 and represented by arbitrary units.

Using a regression analysis of neural activity during the treatment condition, we probed brain regions with neural activity associated with CARE scores, specifically in networks related to empathy for pain and compassion. The regression analysis revealed significant relations between higher CARE scores and activity during treatment in the mOFC/sgACC (*x* = 9, *y* = 41, *z* = −17; *t*-value = 3.76; FWE-corrected *p*-value (voxel-level) = 0.013; FWE-corrected *p*-value (cluster-level) = 0.046) and rACC/vmPFC (*x* = −15, *y* = 38, *z* = 10; *t*-value = 3.64; FWE-corrected *p*-value (voxel-level) = 0.015; FWE-corrected *p*-value (cluster-level) = 0.029)) (Fig. 2).

## Discussion

The neural correlates of positive patient-clinician interactions are largely unknown. Here, we report novel findings from a unique neuroimaging study, where physician’s brains were scanned during face-to-face interaction with a patient^15^. In addition to the primary results from our previous publication^15^, results suggest that a patient’s subjective experience of a physician (CARE rating) after a medical examination was closely linked both to physician empathic concern (assessed by EC subscale of IRI) and treatment-related activations of brain areas associated with reward and compassion in the medial prefrontal cortex (including mOFC/sgACC and rACC/vmPFC)^1^. However, CARE was not associated with distressful empathy responses related to the mirroring of negative feelings of others or activations in the empathy for pain network (anterior insula and caudal anterior cortex). Our results therefore suggest that compassion and reward processing is more consistently engaged than distressful empathy in successful patient–physician interactions.

The relationship between a patient and treating clinician is often described as something intangible and out of reach of systematic scientific investigation. Yet, recent advances in cognitive and social neuroscience have provided a research avenue, where the autonomic and neural processes associated with successful social communication and different aspects of empathy can be determined^6^. Previously, behavioral studies have suggested a shared physiology between patients and physicians, via synchronized autonomic responses during consultations^19^. Also, such measurements suggest that patients’ ratings of physician empathy correlate to degree of autonomic synchronization^20^, indicating that objective markers may be helpful in training and improvement of relational empathy in clinical practice. Here, we were able to disentangle physician personal distress and empathic concern and elucidate how they relate to patients’ subjective experience as captured by quantitative ratings, combined with analyses of physician brain activation patterns.

The IRI instrument^8^ was used to estimate physicians’ self-rated interpersonal sensitivity on two distinct components. The EC dimension reflects tender feelings to others in need, and is considered to be equivalent of compassion^2,3^, whereas the PD dimension (personal distress) reflects distressful components of empathy and one’s own anxiety and feelings of unease when others are in need of help. The important distinction is whether the interaction evokes mainly emotionally positive or distressful reactions in the physician. Data from the physicians in our study indicate that the positive component is central for successful clinical interactions.

Both insula and caudal ACC are core components in processing of empathy for pain^12^. Insula has been linked to feeling states based on interoceptive processing^21^ and often overlapping activation is observed for pain and empathy for pain processes, although the empathy processing is generally more anterior^12^ and includes some unique activation patterns^22^. In the first reports from this dataset, we observed activations in empathy for pain related regions such as the anterior insula during a no treatment condition, where physicians were not able to provide analgesia to their patient^15^. The fact that there was no detectable link between CARE and insular activation during the treatment condition suggests that the negative affect component of empathy reflected in the insula may not be a major factor for successful patient–physician interactions.

Similarly to the insula, caudal ACC may also be activated during evoked pain and processing of empathy for pain. During pain, the caudal ACC has been associated with motor activity, such as avoidance behavior^23^. We did not observe any activation of the caudal ACC during treatment (treatment vs. no treatment)^15^. In fact, lower activations in empathy for pain related regions have been observed in physicians when viewing subjects in pain, as compared to non-physicians^24^. Thus, these studies suggest that physicians suppress aversive empathy-related responses during treatment.

In contrast to the regions processing negative affective components of pain and empathy (discussed above), medial frontal regions (sgACC/mOFC and rACC/vmPFC) were significantly related to CARE scores. These sets of regions have been related to the subjective experience of reward. While rACC/vmPFC has been associated with subjective reward value^25^ and the added value used in value-based decisions^26,27^ the mOFC has been linked to hedonic processing^28^. Thus, we argue that our findings of a positive link between CARE scores and activation in these medial brain regions point to a central role of reward and subjective feelings of pleasantness in patient–physician interactions. In contrast, we did not observe any relation between CARE and ventral striatum, a region involved in processing reward related error responses. Possibly, the present study is inadequately powered to detect such a relation, but alternatively, cortical reward processes may be of more significance in relation to ratings of the patient’s experience of the physician’s social interaction.

Although the present study gives support for compassion related processes being related to successful patient–clinician interactions, the personal distress component of empathy may play a significant role as well. It has been suggested that such distressful processes are important for the motivational drive to help someone in need, and activation of anterior insula when viewing others that suffer may have an important impact on decisions relating to helping others^29,30^. Thus, such processes may be expected before a decision to treat is taken rather than during the treatment itself.

One limitation of this study is the one-sided neuroimaging of physicians that prevents any analyses of neural synchronization between the patient and physician. Recent advances in neuroimaging techniques have allowed for so-called hyperscanning, where two people can be scanned at the same time in two parallel scanners. Such data will be able to increase our understanding of the dynamics of compassion-related brain activations in patients and clinicians. Yet, our experiment allowed the patient to sit face to face with the physician in the scanner room, creating a physical closeness that will not be feasible when two individuals are placed in two separate scanners. Another limitation is the small sample of physicians used in this study, which means that our results should mainly be seen as the first indication of a novel research line where the neural correlates of successful clinical encounters can be assessed in larger samples. Furthermore, the correlational nature of the study prevents causal conclusions about factors that contribute to successful patient–clinician interactions.

In summary, our results suggest that although the distressful component of empathy may be of importance in the motivational drive to treat, reward processes and compassion are more related to a successful patient–physician interaction. Thus—apart from clinicians’ medical skills and knowledge—compassionate caring for someone may be a determinate of a successful treatment.
